# Nutritional index in relation to prognosis of endometrial cancer

**DOI:** 10.7150/ijms.87752

**Published:** 2024-01-01

**Authors:** Yuki Gen, Jisu Yun, Jimin Ahn, Joo Hee Yoon, Dong Choon Park, Sang Il Kim

**Affiliations:** 1 Department of Obstetrics and Gynecology, St. Vincent's Hospital, College of Medicine, The Catholic University of Korea, Seoul, Republic of Korea.; 2 Department of Obstetrics and Gynecology, Eunpyeong St. Mary's Hospital, College of Medicine, The Catholic University of Korea, Seoul, Republic of Korea.

**Keywords:** endometrial cancer, uterine cancer, prognostic factors, prognostic nutritional index, PNI

## Abstract

**Objective:** Evaluate the prognostic value of the prognostic nutritional index (PNI) in patients with endometrial cancer (EC).

**Method:** Laboratory and clinicopathological data from 370 patients who were diagnosed with EC between January 2010 and December 2021 were reviewed. The PNI was analyzed for correlations with recurrence and survival. The receiver operating characteristic curves were generated for the PNI. Optimal cut-off values were determined as the points at which the Youden index (sensitivity + specificity - 1) was maximal. Based on the results of the ROC curve analysis, the patients were grouped into high and low PNI groups. Differences in the clinicopathological characteristics between patients with high and low PNI were compared between the two groups. The effects of the prognostic factors were analyzed using univariate and multivariate Cox proportional hazards model.

**Results:** The optimal cutoff value of the PNI was 52.74 for DFS (area under the curve: 0.817; 95% CI: 0.738-0.858, *p* <0.001). Significantly more patients in the low PNI group experienced recurrence (30.6% vs. 5.2%, *p* <0.001) and cancer-related death (17.8% vs. 2.8%, *p* <0.001). In multivariate analysis, PNI were independent prognostic factors for both DFS and overall survival OS.

**Conclusion:** Low PNI was significantly associated with worse clinical outcomes in patients with EC. Our findings demonstrate that the PNI may be clinically reliable and useful as a prognostic marker for patients with EC. Further large-scale prospective studies are needed to confirm our findings.

## Introduction

Endometrial cancer (EC) is the most common gynecological cancer in developed countries 1. It affects more than 400,000 women annually worldwide, and its incidence is increasing 2. Approximately 66,200 new cases and 13,030 deaths related to EC are expected to occur in the United States by 2023 3. In Korea, EC incidence is increasing, and approximately 3,813 new cases and 445 deaths related to EC are expected to occur by 2023 4. Approximately 70% patients with EC are diagnosed with stage I disease that is surgically curable, leading to nearly 90% of 5-year survival rates 5. However, patients with advanced or recurrent disease have poor prognosis. Owing to the lack of curative treatment options, the 5-year survival rate of patients with advanced or recurrent EC is less than 20% 6.

Classical prognostic factors for EC are well established. These factors include age, stage, grade, histologic subtype, tumor size, lymphovascular space invasion (LVSI), and myometrial invasion (MMI) 7, 8. However, these conventional risk factors are not sufficiently accurate in predicting survival outcomes. Thus, identifying new prognostic factors is crucial to detect high-risk patients during pre-treatment assessments.

Recent studies have supported the importance of nutritional and immunological status in carcinogenesis, progression, and prognosis 9, 10. The effect of pre-operative immuno-nutritional status on survival outcomes has been explored in numerous solid malignancies 11-15. The prognostic nutritional index (PNI) reflects the immuno-nutritional status of patients with cancer, estimated based on pre-operative lymphocyte counts and serum albumin levels 16. The PNI has been widely used to predict the prognosis of gynecologic cancer 17-20. However, EC remains relatively understudied compared to ovarian and cervical cancers 21. To the best of our knowledge, the prognostic value of PNI in patients with EC is unclear. Therefore, this study aimed to evaluate the prognostic value of PNI in patients with EC.

## Materials and Methods

This retrospective, single center study was approved by the Institutional Review Board of the Catholic University of Korea (VC23RASI0257). The requirement for informed consent was waived because of the retrospective nature of the study. This study was conducted in accordance with the principles of the Declaration of Helsinki.

We reviewed our institution's cancer registry and identified patients diagnosed with EC between January 2010 and December 2021. The medical records were retrospectively reviewed. Data of 385 patients were recorded in a single database. We excluded patients who refused to receive treatment in accordance with international guidelines; those with a history of inflammatory, hematological, or autoimmune diseases; those with no laboratory analysis performed within 1 week before treatment; or those with incomplete clinicopathological data or follow-up information. Finally, 370 patients were included in this study.

The primary treatment for most patients was surgery, including total hysterectomy, bilateral salpingo-oophorectomy, and systematic lymphadenectomy. Systemic lymphadenectomy includes pelvic and para-aortic lymphadenectomies. Postoperatively, the patients underwent adjuvant therapy, according to the disease risk factors and international guidelines 22, 23. A few women with distant metastases received primary chemotherapy, with or without delayed surgery.

Laboratory tests, including complete blood cell counts and serum albumin levels, were performed for all patients. PNI was defined as 10 × serum albumin level (g/dL) + 0.005 × absolute lymphocyte count 16. Disease-free survival (DFS) was measured from the date of EC diagnosis to the date of the first recurrence. If the patient had no recurrence, DFS was measured from the date of EC diagnosis to the date of death or last follow-up. Overall survival (OS) was measured from the date of EC diagnosis to the date of cancer-related death or last follow-up. The primary and secondary endpoints were DFS and OS, respectively.

Receiver operating characteristic (ROC) curves of DFS were generated for PNI. The optimal cut-off values of the PNI were determined as the points at which the Youden index (sensitivity + specificity - 1) was maximal. Based on the results of the ROC curve analysis, the patients were grouped into high and low PNI groups. We assessed differences in the clinicopathological characteristics between patients with high and low PNI. Fisher's exact test and chi-square test were used to compare categorical variables. Continuous variables were compared using Student's t-test or Mann-Whitney test. Survival curves for DFS and OS were analyzed using the Kaplan-Meier method, and differences were compared using the log-rank test. We performed univariate and multivariate analyses using the Cox proportional hazards model to analyze the effects of the prognostic factors. All statistical analyses were performed using the Statistical Package for the Social Science (SPSS) statistical software package (version 22.0; SPSS Inc., Chicago, IL, USA). Statistical significance was set at P <0.05.

## Results

Overall, 370 patients were included in the final analysis. The baseline patient characteristics are presented in Table 1. The median age at diagnosis was 56 years (range, 27-86 years). Stage I, II, III, and IV was observed in 274 (74.1%), 16 (4.3%), 60 (16.2%), and 20 (5.4%) patients, respectively. Forty (10.8%) patients had a high-risk non-endometrioid histology. LVSI and lymph node metastasis was observed in 81 (21.9%) and 38 (10.2%) patients, respectively. In total, 180 (48.6%) patients received adjuvant therapy, 112 (30.2%) received radiotherapy, and 68 (18.4%) received chemotherapy. During a median observation period of 45 months (range: 1-159 months), 59 (15.9%) patients experienced tumor recurrence and 34 (9.2%) died of cancer-related causes.

**Figure 1:**
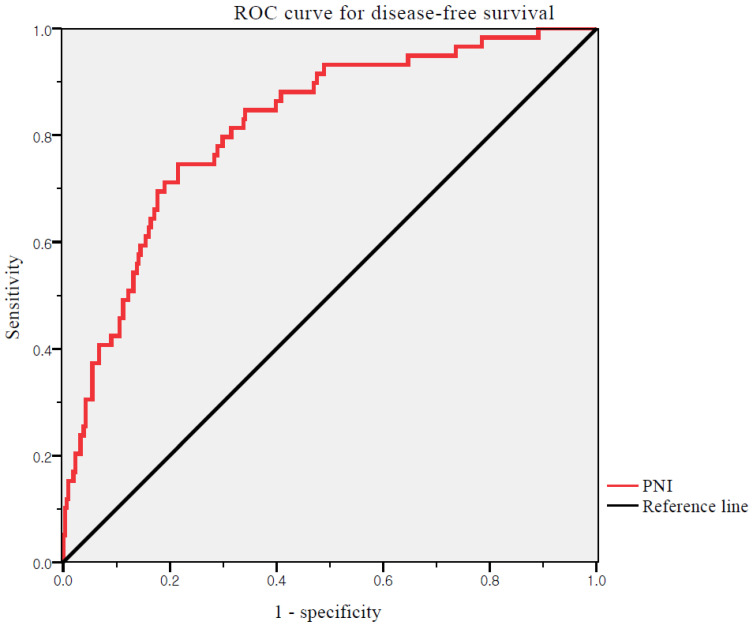
ROC curves for DFS of PNI. Optimal PNI cut-off value was 52.74. The AUC was 0.817. ROC, receiver operating characteristic; PNI, prognostic nutritional index; AUC, area under the curve

We used ROC curve analysis to define the thresholds of the PNI (Figure 1). The median PNI level was 53.68 (range 29.47-69.27). The optimal cutoff value of the PNI was 52.74 for DFS (area under the curve: 0.817; 95% CI: 0.738-0.858, *p* <0.001). The differences in the PNI scores were statistically significant. Thus, the PNI cutoff was used to divide patients into high (PNI ≥52.74) and low (PNI <52.74) PNI groups.

The associations between clinicopathological factors and the PNI are shown in Table 2. The low and high PNI groups included 157 (42.4%) and 213 (57.6%) patients, respectively. No statistically significant differences were observed between the two groups in terms of age and BMI. The two groups differed significantly in the following categorical variables: FIGO stage (*p* = 0.007), histological grade and type (*p* = 0.015, *p* = 0.043), MMI (*p* = 0.001), tumor size (*p* = 0.002), LVSI (*p* = 0.001), LN metastasis (*p* = 0.004), and administration of adjuvant therapy (*p* = 0.033). Significantly more patients experienced recurrence (30.6% vs. 5.2%, *p* <0.001) and cancer-related death (17.8% vs. 2.8%, *p* <0.001) in the low PNI group than in the high PNI group.

Cox proportional hazards model was used to evaluate prognostic factors for DFS and OS (Table 3). In the multivariate analysis, histological grades 2 and 3, MMI, LN metastasis, adjuvant radiotherapy, and high PNI were independent prognostic factors for DFS; whereas advanced stage, histological grade 3, adjuvant radiotherapy, chemotherapy, and high PNI were independent prognostic factors for OS. Consequently, histological grade 3, adjuvant radiotherapy, and a high PNI were independent prognostic factors for both DFS and OS.

**Figure 2:**
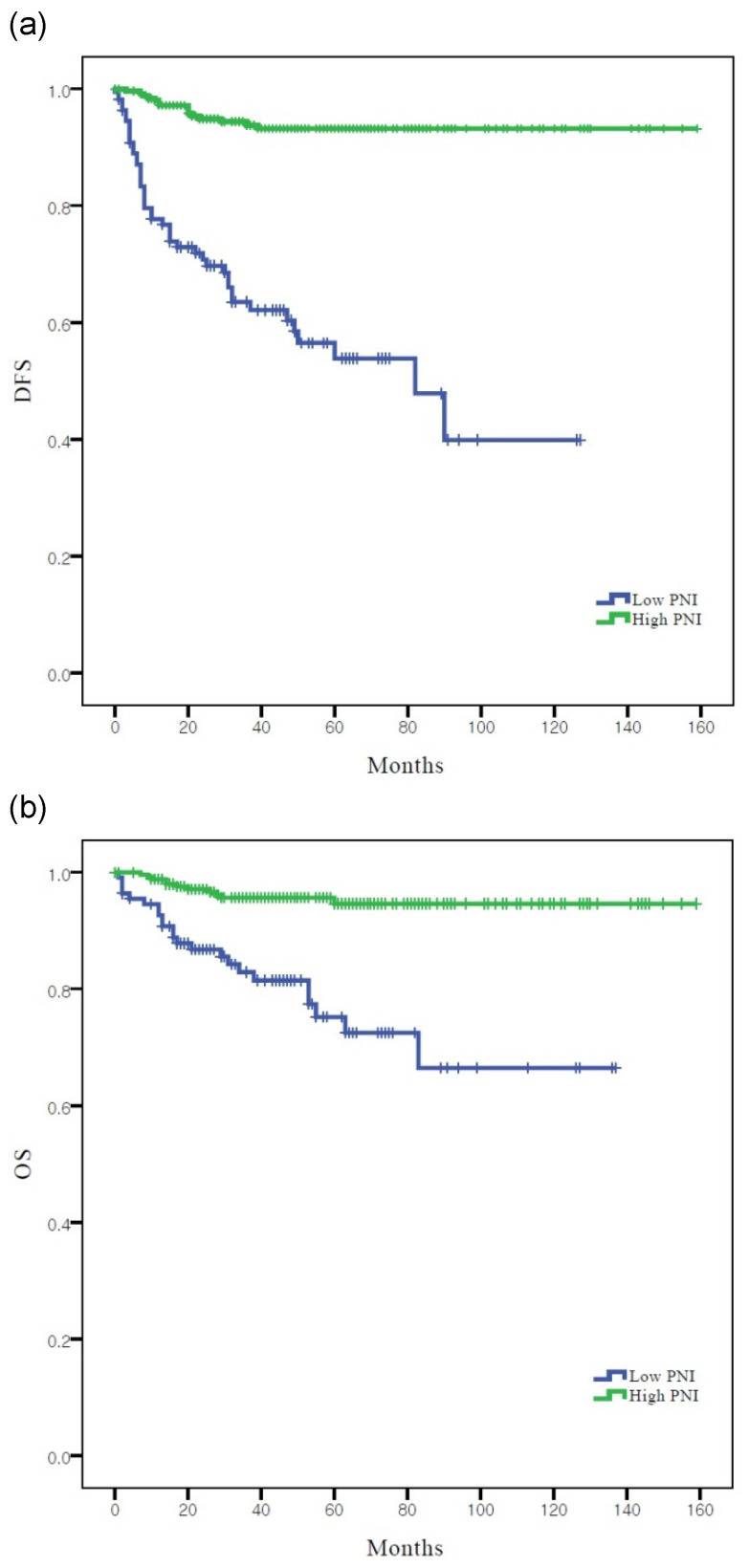
Survival curves according to PNI: (**a**) Kaplan-Meier survival curves for DFS of patients. (**b**) Kaplan-Meier survival curves for OS of patients. DFS, disease-free survival; OS, overall survival; PNI, prognostic nutritional index

According to Kaplan-Meier analysis, the 5-year DFS rates in the low and high PNI groups were 53.8% and 93.2% (log-rank *p* <0.001), respectively, and the 5-year OS rates in these two groups were 81.5% and 96.2%, respectively (log-rank *p* = 0.001) (Figure 2). Both DFS and OS rates were significantly better in the high PNI group than in the low PNI group.

## Discussion

The abnormal nutritional and immunologic status is more likely to decrease the response to anti-tumor therapy and contribute to tumor growth and progression 24, 25. The PNI reflects both the nutritional and immunologic status of patients with cancer.

The PNI was first described by Buxby et al. in 1980 26. It was initially used to estimate the risk of postoperative complications according to the baseline nutritional status. This index provided an accurate, quantitative estimate of operative risk, permitting rational selection of patients who were malnourished and preoperatively treated with nutritional support 16, 26. Since then, numerous studies have highlighted the importance of PNI, not only for postoperative complications but also for the prognosis of solid tumors, including gynecologic cancer 27, 28. However, compared with ovarian and cervical cancers, EC remains relatively understudied. A meta-analysis by Wang et al. showed that the PNI was significantly associated with DFS and OS in patients with cervical and ovarian cancers; however, studies on EC were not included 21.

In this study, we found that the preoperative PNI was an independent predictor of both DFS and OS in patients with EC. A lower PNI was associated with shorter DFS and OS. These results are in concordance with those of previous studies that suggested that the PNI is associated with survival in patients with colorectal, lung, breast, and gastric cancers 29-32. In addition, a low PNI was associated with other traditional prognostic factors, such as FIGO stage, histological grade and type, MMI, tumor size, LVSI, and LN metastasis.

Our results indicated that the PNI is associated with survival in patients with EC, suggesting that immuno-nutritional status is important in this disease. It may be possible to identify patients who are at high risk of recurrence or death after the standard treatment. As PNI is based on serum albumin levels and absolute peripheral lymphocyte counts, routinely measured before surgery, we can assume that patients with EC with a low PNI can benefit from nutritional support before surgery. However, clinical trials on this topic are lacking. Well-designed, large-scale, randomized controlled clinical trials are required to confirm the value of preoperative management in patients with a low PNI.

The mechanisms underlying the association between low PNI and poor outcomes remain unclear. Various studies have reported the anti-tumor functions of lymphocytes 33, 34. Lymphocytes are the main effectors of the immune system that clear tumors from the body and prevent their development and spread 35. CD8^+^ T lymphocytes play a vital role in the immune response against tumor growth, and tumor-specific antigen recognition by them allows malignant cell killing 36. CD4^+^ T lymphocytes produce several inflammatory cytokines that can elicit a vigorous anti-tumor immune response 37. Serum albumin levels reflect a patient's nutrition status 38. Further, malnutrition is related to impaired immune function and poor prognosis in patients with malignant tumors 39, 40. As both lymphocytes and albumin are related to the immune system, low serum albumin levels and low lymphocyte counts may be associated with a poor prognosis. Thus, a low PNI may be a prognostic marker for impaired immune function, leading to tumor growth, progression, and metastasis.

This study had certain limitations. First, this was a retrospective study performed at a single institution and included a limited number of patients. Second, the PNI is a non-specific tumor marker, and there is no defined PNI value for patients with ECs. We set a cut-off value for our study. Third, although we calculated the PNI based on laboratory tests performed within 1 week before surgery, the PNI might have been affected by various conditions, and it varies from time to time. Further large-scale multicenter prospective studies will provide more definitive data to confirm the results of our study.

In conclusion, our findings demonstrate that the PNI may be clinically reliable and useful as a prognostic marker for patients with EC. The PNI, along with many clinicopathological features, was significantly associated with DFS and OS. Further large-scale prospective studies are needed to confirm our findings and identify appropriate cutoff values.

## Data Availability

The data that support the findings of this study are available on request from the corresponding author.

## Figures and Tables

**Table 1:** Baseline patient characteristics (n = 370)

	No. of patients	%
Age (years), median (range)	56 (27 - 86)	
BMI (kg/m2), median (range)	24.79 (15.22 - 39.27)	
Surgical approach		
Open	123	33.2
MIS*	247	66.8
FIGO stage		
I	274	74.1
II	16	4.3
III	60	16.2
IV	20	5.4
Grade		
1	162	43.8
2	121	32.7
3	87	23.5
Histology		
Endometrioid	330	89.2
Non-endometrioid+	40	10.8
MMI		
< 50%	252	68.1
≥ 50%	118	31.9
Tumor size (cm) , mean ± SD	3.57 ± 2.78	
LVSI		
Absent	289	78.1
Positive	81	21.9
LN metastasis		
Absent	332	89.8
Positive	38	10.2
Adjuvant therapy		
None	190	51.4
Radiotherapy	112	30.2
Chemotherapy	68	18.4
Follow-up (months), median (range)	45 (1 - 159)	
Overall recurrences	59	15.9
Deaths	34	9.2

^*^both conventional laparoscopy and robot assisted laparoscopy, ^+^serous, clear, carconosarcoma, undifferentiated, dedifferentiated BMI, body mass index; MIS, minimally invasive surgery; FIGO, International Federation of Gynecology and Obstetrics; MMI, myometrial invasion; SD, standard deviation; LVSI, lymphovascular space invasion; LN, lymph node

**Table 2:** Clinico-pathological characteristics according to the PNI (n=370)

	Low PNI group (n = 157, %)	High PNI group (n = 213, %)	*p* value
Age (years), median (range)	56 (27 - 86)	55 (30 - 81)	0.837
BMI (kg/m^2^), median (range)	23.8 (15.2 - 39.3)	25.2 (17.8 - 38.5)	0.053
FIGO stage			0.007^+^
I	103 (65.6)	170 (80.3)	
II	8 (5.1)	8 (3.8)	
III	32 (20.4)	28 (13.1)	
IV	14 (8.9)	6 (2.8)	
			
Grade			0.015^+^
1	57 (36.3)	105 (49.3)	
2	53 (33.8)	68 (31.9)	
3	47 (29.9)	40 (18.8)	
			
Histology			0.043^+^
Endometrioid	134 (85.4)	196 (92.0)	
Non-endometrioid^*^	23 (14.6)	17 (8.0)	
			
MMI			0.001^+^
< 50%	91 (58.0)	161 (75.6)	
≥ 50%	66 (42.0)	52 (24.4)	
			
Tumor size (cm), mean ± SD	4.12 ± 3.28	3.17 ± 2.28	0.002^+^
			
LVSI			0.001^+^
Absent	109 (69.4)	180 (84.5)	
Positive	48 (30.6)	33 (15.5)	
			
LN metastasis			0.004^+^
Absent	133 (84.7)	199 (93.4)	
Positive	24 (15.3)	14 (6.6)	
			
Adjuvant therapy			0.033^+^
None	70 (44.6)	120 (56.3)	
Radiotherapy	47 (29.9)	65 (30.5)	
Chemotherapy	40 (25.5)	28 (13.1)	

^*^serous, clear, carconosarcoma, undifferentiated, dedifferentiated, ^+^*p* value < 0.05 PNI, prognostic nutritional index; BMI, body mass index; FIGO, International Federation of Gynecology and Obstetrics; MMI, myometrial invasion; SD, standard deviation; LVSI, lymphovascular space invasion; LN, lymph node

**Table 3:** Multivariate analysis of prognostic factors for disease-free survival and overall survival (n = 370)

Characteristics	Multivariate analysis for DFS			Multivariate analysis for OS		
	OR	95% CI	*p* value	OR	95% CI	*p* value
**Age**	1.018	0.991 - 1.045	0.204	0.985	0.952 - 1.020	0.985
**BMI**	1.005	0.941 - 1.073	0.889	0.965	0.880 - 1.057	0.965
**FIGO stage**						
**I**	1 (Ref)	-	-	1 (Ref)	-	-
**II**	3.457	0.885 - 13.50	0.074	9.415	0.805 - 110.1	0.074
**III**	1.632	0.501 - 5.315	0.416	13.99	3.386 - 57.80	0.001^+^
**IV**	2.180	0.545 - 8.710	0.270	38.23	8.646 - 169.0	0.001^+^
**Grade**						
**1**	1 (Ref)	-	-	1 (Ref)	-	-
**2**	4.589	1.745 - 12.07	0.003^+^	2.207	0.471 - 10.35	0.315
**3**	5.916	1.901 - 18.41	0.002^+^	12.208	2.832 - 52.63	0.001^+^
**Histology**						
**Endometrioid**	1 (Ref)	-	-	1 (Ref)	-	-
Non-endometrioid^*^	2.014	0.921 - 4.401	0.136	1.778	0.724 - 4.366	0.209
**MMI**						
**< 50%**	1 (Ref)	-	-	1 (Ref)	-	-
**≥ 50%**	3.494	1.434 - 8.510	0.017^+^	1.440	0.429 - 4.837	0.555
**Tumor size**	1.130	1.022 - 1.250	0.084	1.059	0.932 - 1.203	0.378
**LVSI**						
**No**	1 (Ref)	-	-	1 (Ref)	-	-
**Yes**	1.420	0.548 - 3.677	0.470	1.475	0.430 - 5.062	0.536
**LN metastasis**						
**No**	1 (Ref)	-	-	1 (Ref)	-	-
**Yes**	2.265	1.118 - 4.587	0.023^+^	2.123	0.800 - 5.632	0.131
**Adjuvant therapy**						
**None**	1 (Ref)	-	-	1 ( Ref)	-	-
**Radiotherapy**	0.159	0.058 - 0.438	0.001^+^	0.174	0.038 - 0.800	0.025^+^
**Chemotherapy**	0.386	0.135 - 1.105	0.076	0.142	0.030 - 0.678	0.014^+^
**PNI**						
**< 52.74**	1 (Ref)	-	-	1 (Ref)	-	-
**≥ 52.74**	0.156	0.080 - 0.326	< 0.001^+^	0.266	0.105 - 0.673	0.005^+^

^*^serous, clear, carconosarcoma, undifferentiated, dedifferentiated, ^+^*p* value < 0.05 DFS, disease-free survival; OS, overall survival; OR, odds ratio; CI, confidence interval; Ref, reference; BMI, body mass index; FIGO, International Federation of Gynecology and Obstetrics; LVSI, lymphovascular space invasion; LN, lymph node; PNI, prognostic nutritional index

## References

[1] Sung H, Ferlay J, Siegel RL (2021). Global Cancer Statistics 2020: GLOBOCAN Estimates of Incidence and Mortality Worldwide for 36 Cancers in 185 Countries. CA Cancer J Clin.

[2] Crosbie EJ, Kitson SJ, McAlpine JN, Mukhopadhyay A, Powell ME, Singh N (2022). Endometrial cancer. Lancet.

[3] Siegel RL, Miller KD, Wagle NS, Jemal A (2023). Cancer statistics, 2023. CA Cancer J Clin.

[4] Jung KW, Kang MJ, Park EH (2023). Prediction of Cancer Incidence and Mortality in Korea, 2023. Cancer Res Treat.

[5] Sorosky JI (2012). Endometrial cancer. Obstet Gynecol.

[6] Lee YC, Lheureux S, Oza AM (2017). Treatment strategies for endometrial cancer: current practice and perspective. Curr Opin Obstet Gynecol.

[7] Hecht JL, Mutter GL (2006). Molecular and pathologic aspects of endometrial carcinogenesis. J Clin Oncol.

[8] Morice P, Leary A, Creutzberg C, Abu-Rustum N, Darai E (2016). Endometrial cancer. Lancet.

[9] Mellman I, Coukos G, Dranoff G (2011). Cancer immunotherapy comes of age. Nature.

[10] Zitvogel L, Pietrocola F, Kroemer G (2017). Nutrition, inflammation and cancer. Nat Immunol.

[11] Nie R, Yuan S, Chen S (2016). Prognostic nutritional index is an independent prognostic factor for gastric cancer patients with peritoneal dissemination. Chin J Cancer Res.

[12] Li S, Tian G, Chen Z, Zhuang Y, Li G (2019). Prognostic Role of the Prognostic Nutritional Index in Pancreatic Cancer: A Meta-analysis. Nutr Cancer.

[13] Man Z, Pang Q, Zhou L (2018). Prognostic significance of preoperative prognostic nutritional index in hepatocellular carcinoma: a meta-analysis. HPB (Oxford).

[14] Sun G, Li Y, Peng Y (2019). Impact of the preoperative prognostic nutritional index on postoperative and survival outcomes in colorectal cancer patients who underwent primary tumor resection: a systematic review and meta-analysis. Int J Colorectal Dis.

[15] Chen L, Bai P, Kong X (2021). Prognostic Nutritional Index (PNI) in Patients With Breast Cancer Treated With Neoadjuvant Chemotherapy as a Useful Prognostic Indicator. Front Cell Dev Biol.

[16] Onodera T, Goseki N, Kosaki G (1984). [Prognostic nutritional index in gastrointestinal surgery of malnourished cancer patients]. Nihon Geka Gakkai Zasshi.

[17] Haraga J, Nakamura K, Omichi C (2016). Pretreatment prognostic nutritional index is a significant predictor of prognosis in patients with cervical cancer treated with concurrent chemoradiotherapy. Mol Clin Oncol.

[18] Zhang W, Ye B, Liang W, Ren Y (2017). Preoperative prognostic nutritional index is a powerful predictor of prognosis in patients with stage III ovarian cancer. Sci Rep.

[19] Ida N, Nakamura K, Saijo M, Kusumoto T, Masuyama H (2018). Prognostic nutritional index as a predictor of survival in patients with recurrent cervical cancer. Mol Clin Oncol.

[20] Feng Z, Wen H, Ju X (2018). The preoperative prognostic nutritional index is a predictive and prognostic factor of high-grade serous ovarian cancer. BMC Cancer.

[21] Wang X, Wang Y (2019). The prognostic nutritional index is prognostic factor of gynecological cancer: A systematic review and meta-analysis. Int J Surg.

[22] Koh WJ, Abu-Rustum NR, Bean S (2018). Uterine Neoplasms, Version 1.2018, NCCN Clinical Practice Guidelines in Oncology. J Natl Compr Canc Netw.

[23] Colombo N, Preti E, Landoni F (2013). Endometrial cancer: ESMO Clinical Practice Guidelines for diagnosis, treatment and follow-up. Ann Oncol.

[24] Liu X, Sun X, Liu J (2015). Preoperative C-Reactive Protein/Albumin Ratio Predicts Prognosis of Patients after Curative Resection for Gastric Cancer. Transl Oncol.

[25] Okadome K, Baba Y, Yagi T (2020). Prognostic Nutritional Index, Tumor-infiltrating Lymphocytes, and Prognosis in Patients with Esophageal Cancer. Ann Surg.

[26] Buzby GP, Mullen JL, Matthews DC, Hobbs CL, Rosato EF (1980). Prognostic nutritional index in gastrointestinal surgery. Am J Surg.

[27] Takushima Y, Abe H, Yamashita S (1994). [Evaluation of prognostic nutritional index (PNI) as a prognostic indicator in multimodal treatment for gynecological cancer patients]. Gan To Kagaku Ryoho.

[28] Komura N, Mabuchi S, Yokoi E (2019). Prognostic significance of the pretreatment prognostic nutritional index in patients with epithelial ovarian cancer. Oncotarget.

[29] Tominaga T, Nagasaki T, Akiyoshi T (2020). Prognostic nutritional index and postoperative outcomes in patients with colon cancer after laparoscopic surgery. Surg Today.

[30] Li D, Yuan X, Liu J, Li C, Li W (2018). Prognostic value of prognostic nutritional index in lung cancer: a meta-analysis. J Thorac Dis.

[31] Hua X, Long ZQ, Huang X (2019). The Value of Prognostic Nutritional Index (PNI) in Predicting Survival and Guiding Radiotherapy of Patients With T1-2N1 Breast Cancer. Front Oncol.

[32] Xishan Z, Ye Z, Feiyan M, Liang X, Shikai W (2020). The role of prognostic nutritional index for clinical outcomes of gastric cancer after total gastrectomy. Sci Rep.

[33] Yu P, Fu YX (2006). Tumor-infiltrating T lymphocytes: friends or foes?. Lab Invest.

[34] Caruana I, Simula L, Locatelli F, Campello S (2018). T lymphocytes against solid malignancies: winning ways to defeat tumours. Cell Stress.

[35] Hanahan D, Weinberg RA (2011). Hallmarks of cancer: the next generation. Cell.

[36] Hadrup S, Donia M, Thor Straten P (2013). Effector CD4 and CD8 T cells and their role in the tumor microenvironment. Cancer Microenviron.

[37] Li T, Wu B, Yang T, Zhang L, Jin K (2020). The outstanding antitumor capacity of CD4(+) T helper lymphocytes. Biochim Biophys Acta Rev Cancer.

[38] Fuhrman MP, Charney P, Mueller CM (2004). Hepatic proteins and nutrition assessment. J Am Diet Assoc.

[39] Gupta D, Lis CG (2010). Pretreatment serum albumin as a predictor of cancer survival: a systematic review of the epidemiological literature. Nutr J.

[40] Liu Y, Chen S, Zheng C (2017). The prognostic value of the preoperative c-reactive protein/albumin ratio in ovarian cancer. BMC Cancer.

